# Sir William Overend Priestly

**Published:** 1900-05

**Authors:** 


Sir William Overend Priestly, the distinguished obstetrician, member of the House of Commons for Edinburgh and St. Andrew's Universities, died in London on April 11th. He was born in 1829, and was a grand nephew of Joseph Priestly, the celebrated chemist. He was M. R. C. S. in 1852, M. D. Edin. in 1853, and F.R.C.P. Edin. in 1858.

